# Sorption Capacity of Polydimethylsiloxane Foams Filled with Thermal-Treated Bentonite—Polydimethylsiloxane Composite Foams for Oil Spill Remediation

**DOI:** 10.3390/ma16134818

**Published:** 2023-07-04

**Authors:** Luigi Calabrese, Elpida Piperopoulos, Vesna Stankov Jovanović, Jelena Nikolić, Slobodan Ćirić, Candida Milone, Edoardo Proverbio

**Affiliations:** 1 Dipartimento di Ingegneria, Università di Messina, Contra di Dio-Sant’Agata, 98166 Messina, Italy; epiperopoulos@unime.it (E.P.); cmilone@unime.it (C.M.); eproverbio@unime.it (E.P.); 2 Department of Chemistry, Faculty of Science and Mathematics, University of Nis, Visegradska 33, 18 000 Nis, Serbiajelena.cvetkovic7@gmail.com (J.N.);

**Keywords:** oil recovery, foam, siloxane, bentonite, sorption kinetics, oil–water selectivity

## Abstract

The spillage of oil causes severe and long-lasting impacts on both the environment and human life. It is crucial to carefully reconsider the methods and techniques currently employed to recover spilled oil in order to prevent any possible secondary pollution and save time. Therefore, the techniques used to recover spilled oil should be readily available, highly responsive, cost-effective, environmentally safe, and, last but not least, they should have a high sorption capacity. The use of sorbents obtained from natural materials is considered a suitable approach for dealing with oil spills because of their exceptional physical characteristics that support sustainable environmental protection strategies. This article presents a novel sorbent material, which is a composite siloxane foam filled with bentonite clay, aimed at enhancing the hydrophobic and oleophilic behavior of the material. The thermal treatment of bentonite optimizes its sorption capacity by eliminating water, and increasing the surface area, and, consequently, its interaction with oils. In particular, the maximum sorption capacity is observed in kerosene and naphtha for the bentonite clay thermally treated at 600 °C, showing an uptake at saturation of 496.8% and 520.1%, respectively. Additionally, the reusability of the composite foam is evaluated by squeezing it after reaching its saturation point to determine its sorption capacity and reusability.

## 1. Introduction

Over the last twenty years, oil spill pollution has attracted increasing attention due to the widespread damages that can endanger ecosystems and global health. Oil spills in oceans, seas, and other water bodies are a severe environmental threat, causing harm to marine life and ecosystems [1,2]. It is approximated that more than one hundred million gallons of crude oil is spilled into the marine environment yearly [3]. Notable cases include the “Prestige” shipwreck (NA Atlantic) in 2002 [4], the “Sea Diamond” shipwreck (Santorini) in 2007 [5], Deep Horizon (Gulf of Mexico) in 2010 [6], and “Agia Zoni II” (Saronic Gulf) in 2017 [7]. Oil spill remediation is an important issue for several reasons. Oil spills can have a significant impact on the environment and wildlife. Damage that results from oil spills depends mainly on the oil’s chemical structure, affected area and the success of remedial measures [8,9,10]. As oil reaches the surface, its composition and properties change almost immediately. There are a number of processes that take place after an initial oil spillage, including oil evaporation, dissolution, oxidation, emulsification, photo-oxidation, sinking, sedimentation, biodegradation, and dispersion, making remediation more complex [11,12]. Oil disperses in water, forms a slick on the surface, immerses and accumulates in the sediments. Water currents and wind cause a rapid distribution and degradation of oil and harmful effects on the aquatic ecosystem [13,14]. In combination with the aforementioned conditions, a variety of physicochemical parameters such as salinity and temperature determine the oil spills’ spread rates in a natural body of water [15]. When oil spills into waterways, it can harm or kill aquatic plants and animals, disrupt food chains, and cause long-term damage to ecosystems [16]. Furthermore, the toxic chemicals in oil can have negative impacts on human health. Additionally, when oil spills occur, businesses and industries that rely on the affected waterways or coastal areas may suffer financial losses leading to direct or indirect economic consequences [17].

As a result of these impacts, there is an urgent need for effective oil spill remediation methods that can efficiently remove oil from water. Oil spills have occurred for decades, but, unfortunately, remediation techniques have remained largely unchanged over that period of time. These strategies can include containment and cleanup measures, as well as efforts to prevent spills from occurring in the first place, such as improved safety regulations and more sustainable energy practices [18]. Post-oil-spill response techniques include mechanical methods (booms, sorbents, and skimmers), thermal methods (in situ burning), chemical methods (dispersion) and natural methods (bioremediation) [19]. All these methods have advantages and disadvantages; however, the efficacy of each technique often depends on external factors such as oil composition, viscosity and volume, in addition to location and weather conditions. Among these methods and technologies, sorbent materials are more attractive for oil-spill cleanup because of the possibility of collection and complete removal of the oil (which includes water-insoluble organic liquids hereafter) at the site from the water surface, while bringing no adverse effects to the environment. A good sorbent material should have hydrophobic and lyophilic properties, a high oil sorption capacity, and low material costs. Many oil sorbents have been developed using various host materials, including nanoparticles, polymer fabrics, nanowire membranes, metal meshes, polymer sponges and carbon-based porous materials [20]. Traditional oil sorbents, including vegetable materials [21], mineral products, and synthetic materials, experience various problems like low oil–water-separation efficiency, low oil-sorption capacity, and high material costs [22,23]; therefore, the development of novel sorbents is of great interest.

In practice, an ideal sorbent should have the following characteristics: high sorption capacity, hydrophobicity, floating ability before and after sorption process, reusability and low price [24]. Polydimethylsiloxane (PDMS)-based materials are known for their low bulk density, biocompatibility, chemical stability, water resistance, and hydrophobicity [25,26]. The high elasticity and hydrophobicity of PDMS-based sorbents shows excellent recyclability and selectivity compared with that of other previously reported sorbents. The special features of these materials include non-toxicity, low bulk density, biocompatibility, low cost, chemical stability, water resistance, hydrophobicity, and commercial availability [27,28]. Furthermore, to reduce the material cost, a desirable strategy is to use PDMS foams for oil spill pollution as one of the most visible examples of water quality degradation due to anthropogenic activities that affect aquatic ecosystems worldwide [29,30,31]. In a previous work [32], the authors developed bentonite-filled siloxane foams for oil spills’ recovery. Composite foams with filler contents ranging from 35 to 45 wt.% were examined. The sorption kinetics and capacity of composite foams in various oils (for example, kerosene, virgin naphtha, and pump oil) were investigated. The composite foam filled with 40 wt.% bentonite had the lowest affinity with water and the best sorption capacity with oils, with sorption capacities at saturation equal to 10.3 wt.% in water and 518.2 wt.% in virgin naphtha. The high sorption capacity of the composite foam can be attributed to the combined effects of PDMS and bentonite. PDMS provides a porous structure that allows oil to penetrate into the foam, while bentonite provides a high surface area and a high affinity for oil.

Based on these promising results, the development and optimization of a bentonite–PDMS composite foam is a significant step towards the development of effective oil spill remediation methods. The high sorption capacity and reusability of the composite foam could make it a promising candidate for use in oil spill cleanup operations. Therefore, future research focused on optimizing the composition and structure of the composite foam to further enhance its sorption capacity and durability could significantly improve the scientific soundness of this class of materials and its relevance in a critical environmental issue.

The aim of this work is to develop a new type of sorbent material: a composite foam made from polydimethylsiloxane (PDMS) and thermal-treated bentonite in order to enhance the hydrophobicity and oleophilic properties of the material. Bentonite is a clay mineral that has a high affinity for organic compounds such as oil. By incorporating bentonite into PDMS foam, the purpose is to create a material with a high sorption capacity for oil. The thermal treatment of bentonite enhances its sorption capacity by removing water and other impurities and increasing the interaction with oils. The composite foam is finally evaluated by squeezing it after sorption saturation in order to assess its sorption capacity and reusability.

## 2. Experimental Part

### 2.1. Materials

Ethanol (98% purity) and Tin(II) 2-ethyl hexanoate (purity > 92.5%) were acquired from Sigma Aldrich (St. Louis, MO, USA). Polymethylhydrosiloxane, trimethylsilyl-terminated, (PMHS, M.W. 5500–6500) and silanol-terminated polydimethylsiloxane, (PDMS, M.W. 1,100,000), purchased from Gelest Inc. (Morrisville, PA, USA), were used as silicone reactants for the foaming matrix. The natural clay mineral (two silica tetrahedral sheets and one alumina octahedral sheet) selected for PDMS-based sorbent preparation is natural bentonite clay.

Bentonite powder was used as the filler material and it was supplied by “Nemetali” (Vranjska Banja, Serbia). The bentonite clay was thermally treated at different constant temperatures, respectively, 300, 400, 500, 600, 700 and 800 °C for 3 h in an annealing furnace (in static air). Bentonite powder samples were directly put in the oven when the desired temperature was achieved.

The clay structure of bentonite, with silicate and aluminate dominance, is layered, the particles are small and there is inhomogeneity. The particles have different sizes and shapes and are unevenly distributed [33]. The sorbent filler was structurally characterized via X-ray diffraction (XRD), using a D8 Advance Bruker diffractometer (Billerica, MA, USA), in the Bragg–Brentano theta-2ndeta configuration, Cu Kα, 40 V, 40 mA, 0.1°/s scan rate.

### 2.2. Synthesis of the Composite Foams

**Figure 1:**
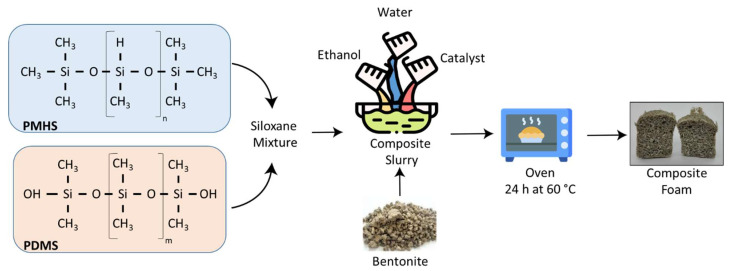
Scheme of the bentonite–silicone foam preparation.

The composite foams were produced according to the procedure reported in [34,35]. The silicone foam was manufactured by using a direct dehydrogenative reaction between siloxane compounds, which was used as a foaming reaction to produce all the composite foams [36]. In particular, trimethylsilyl-terminated Poly(dimethylsiloxane-co-methylhydrosiloxane) (PMHS, M.W. 5500–6500) and silanol-terminated polydimethylsiloxane (PDMS, M.W. 1,100,000) compounds were mixed under magnetic stirring (PDMS/PMHS weight ratio 2:1) for 60 s. Afterward, bentonite powder was progressively added as filler, always under magnetic stirring, until a homogenous slurry was achieved. Furthermore, with the purpose of reducing the viscosity of the composite slurry and to favor physical bubbling, bi-distilled water and ethanol were added into the mixture [36,37]. To activate the dehydrogenative coupling reaction between the siloxane compound, Tin(II)2-ethylhexanoate(Sn(II)) (density: 1.251 g/mL, purity > 92.5%), supplied by Sigma Aldrich (St. Louis, MO, USA), was added as catalyst. Finally, the homogenous composite mixture was poured into a cylindrical aluminum mold (diameter 2 cm, height 5 cm) and placed in an oven (24 h at 60 °C) to activate the foaming process. This latter process is due to the reaction of the hydroxyl and hydrate groups of PDMS and PMHS compounds, respectively, leading to hydrogen gas as the reaction product that acts as a foaming agent [38]. Figure 1 summarizes the synthesis steps of the composite bentonite–silicone foam. Furthermore, Table 1 shows the bentonite–siloxane solution composition of all the composite foams (all percentage are by weight).

All samples were codified with a prefix “BS-” coupled to a number referring to the treatment temperature (expressed in °C) of the bentonite., e.g., BS-300 batch refers to a composite bentonite–silicone foam obtained by using bentonite thermally pre-treated at 300 °C.

### 2.3. Sorption Capacity Tests

Oil sorption tests were performed on squared composite foams portions, with dimensions of about 1 × 1 × 1 cm^3^, attained by cutting cylindrical samples obtained according to procedure reported in Figure 1.

Oil sorption tests were performed, according to [39], at room temperature and under slow stirring in a 250 mL of kerosene, virgin naphtha and pump oil. Furthermore, as a reference, sorption tests in water were also performed for all of the composite foams. The sorption tests were accurately carried out by performing a common measurement protocol for all tests: at soaking time intervals, the sorbent bentonite–PDMS composite sample was prudently pulled out from the beaker. The soaked sample was left to rest in a clean watch glass for 15 s in order to release the excess liquid, which was subsequently weighed. The oil uptake was calculated based on the sample mass variation at increasing soaking time in the oil according to the following expression:

(1) $$Qt=\frac{m_{t}-m_{0}}{m_{0}}\cdot 100$$

where Qt (expressed as wt. %) is the sorption capacity (or the oil uptake) of the foam at the soaking time, and t. m_0_ and m_t_ are the weight of the sample at t = 0 s and at t soaking time, respectively. All sorption measurements were carried out until the oil sorption saturation was achieved (recognizable as the stabilization of the sample weight at increasing soaking time). The experimental protocol for oil sorption capacity was applied to simulate in a laboratory scale the open sea water motion. For this purpose, the test was performed under stirring until weight equilibrium was reached.

Moreover, the foam morphology was investigated using a scanning electron microscope (SEM, FEI Quanta 450, Thermo Fisher Scientific, Waltham, MA, USA), in low vacuum, at an acceleration voltage of 5 kV.

## 3. Results and Discussion

**Figure 2:**
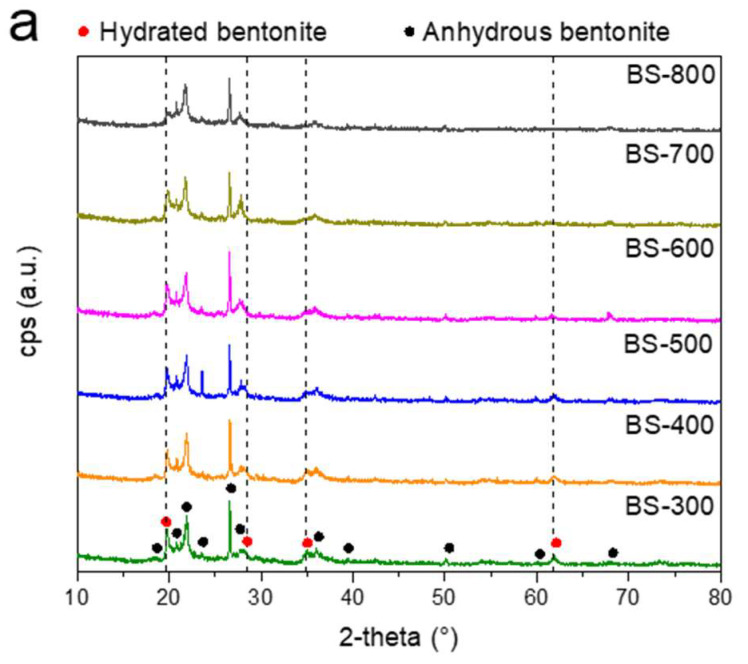

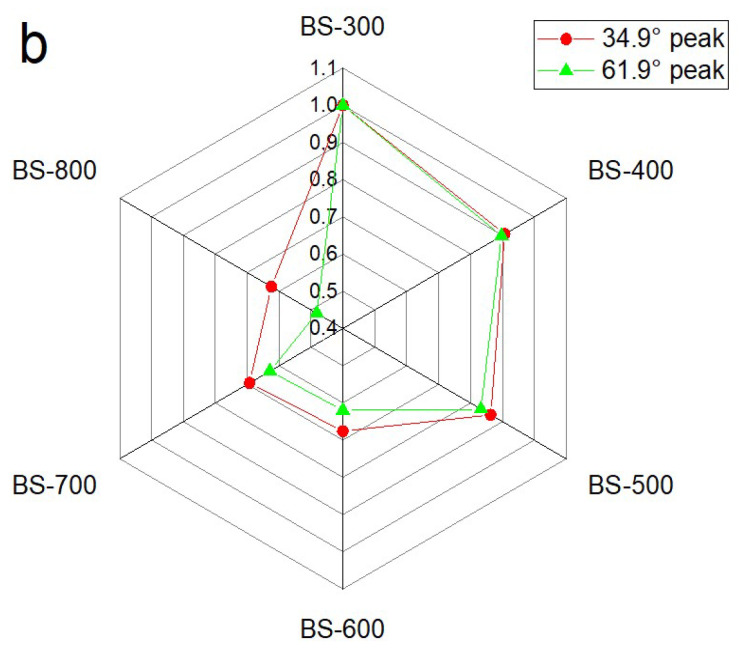
(**a**) XRD analysis of the investigated thermally treated clays and (**b**) polygon plot of the peaks related to the hydrated phase, positioned at 2-theta: 34.9° and 61.9° of the normalized diffractograms.

In Figure 2a, the XRD analysis diffractograms are reported. As evidenced, only bentonite structure is detected, and no impurities are present. The clay is present in its two forms, hydrated (2-theta: 19.7°, 28.2°, 34.9°, 61.9°, PDF-ICDD: 00-001-0026) and anhydrous (2-theta: 20.8°, 21.9°, 26.6°, 27.7°, 35.7°, 39.4°, 50.0°, 59.9°, 68.1, PDF-ICDD: 55-71), respectively. What is important to note is that, when bentonite is treated at 800 °C, clear differences appear in the spectra. In detail, the height of the peak related to the hydrated bentonite at 2-theta, 19.7°, which represents the layered silicate structure of bentonite [40], decreases conspicuously, while the peaks related to the anhydrous bentonite remain clearly evident.

This is probably due to the treatment temperature (800 °C), which enables the loss of crystallization water (dihydroxylation) and the subsequent sintering of the clay [41].

In reality, the dehydration process already occurs gradually at lower temperatures. As depicted in the chart displayed in Figure 2b, the peaks related to the hydrated phase (positioned at 2-theta: 34.9° and 61.9° of the normalized diffractograms) are represented for all the analyzed batches. A gradual reduction in the cps of the peaks, as the treatment temperature increases, is evidenced. Specifically, for both peaks, a substantial intensity reduction is noticeable in the BS-800 sample; even the peak at 19.7° drops significantly, and the shoulder at 28.2° disappears (Figure 2a).

**Figure 3:**
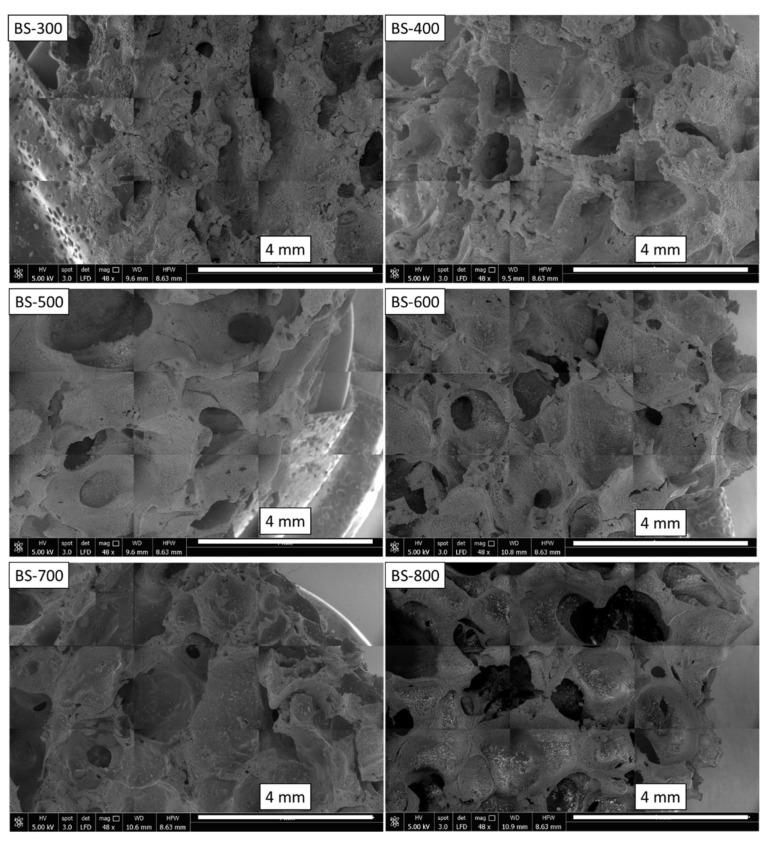
SEM images of investigated foams.

Figure 3 shows the SEM images of all of the synthesized foams. The morphological investigation was carried out to evaluate the distribution and interconnection of the particles and to confirm the interfacial interaction between the matrix and the filler. The silicone foams exhibit a macro-porous structure with quite regular and homogenously distributed bubbles. The pores are interconnected due to bubble agglomeration or microchannels’ pores. The foams exhibit a quite isotropic morphology. Some discrepancies can be identified. Indeed, the SEM investigation also highlighted how bentonite is present on the surface of the foam and consequently has an active role in the sorption mechanisms. This suggests that the clay can act as a filter for oils that come into contact with the foam, which can help improve the overall sorption performance of the material for oil spill recovery.

The average pore size (APS) of the investigated foams, together with the apparent density (ρ_a_) of the composite foam at varying filler typologies, is reported in Table 2. By increasing the temperature of the thermal treatment from 300 °C to 400 °C, an increase in the pore size was detected: 1.02 mm and 1.36 mm, respectively, for BS-300 and BS-400. No evident change in the pore size of the foam was found when we raised the temperature of the bentonite treatment to 600 °C. However, the temperature treatment at 700 °C and 800 °C leads to an enlargement of the pores of the composite foam, reaching 1.83 mm and 2.18 mm, respectively, for BS-700 and BS-800 [42]. Therefore, by increasing the APS, the apparent density decreases; in particular, a 45.2% decrease from sample BS-300 to sample BS-800 was calculated. For samples BS-400, BS-500 and BS-600, the ρ_a_ values remain almost the same. This behavior could be related to the modified surface polarity of the filler that contributes to the chemical foaming process of the PDMS silicone foam, considering that during its dehydrogenative coupling, a reaction hydrogen gas is produced, thereby triggering the foaming process [43]. This can be related to the presence of electronegative groups on the filler surface which, by interacting with the hydride siloxane (PMHS), support the reaction process by stimulating a greater expansion of the foam [37]. As indicated in Table 2, this can be identified from the progressive increase in the size of the pores and the consequent progressive reduction in the apparent density as the temperature of the heat treatment applied to the bentonite increases.

**Figure 4:**
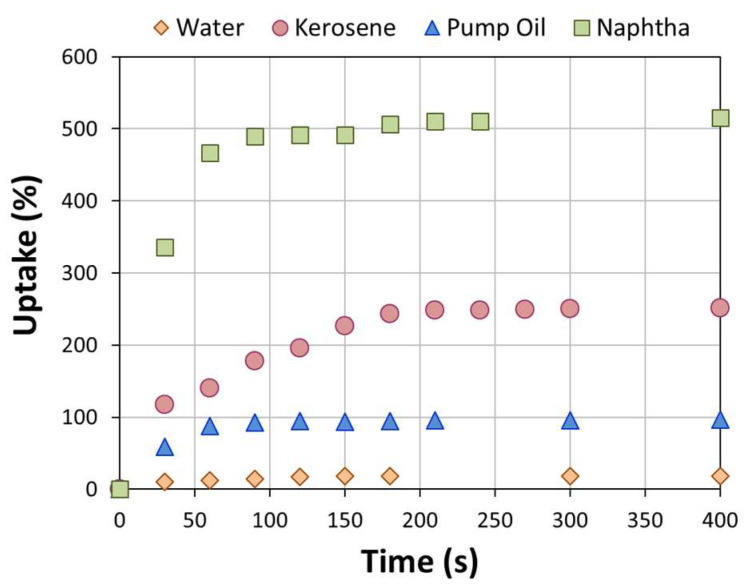
Sorption capacity (uptake) at increasing sorption time in all liquids for BS-300 composite foam.

Figure 4 shows the evolution in the uptake of different liquids during time for a reference BS-300 composite foam. The foam shows a different sorption capacity over time and at different equilibrium states as the liquid varies. The water sorption capacity is very limited, as evidenced by a maximum water sorption at equilibrium equal to 18.1%. The curve has a very flat trend with no significant increases even at high float times. This confirms that the foam has a low surface affinity with water, resulting in a predominantly hydrophobic behavior of the surface.

Conversely, in the presence of hydrocarbons liquids, the foam exhibits a significantly higher uptake. The maximum sorption capacity was found in naphtha, where an uptake to equilibrium equal to 515.7% was acquired. The trend of the uptake curve in this liquid also makes evident that the sorption kinetic is very rapid. In fact, after about 60 s of floating in naphtha, an increase in weight close to that of the equilibrium was acquired. In kerosene and pump oil, a lower sorption capacity was found, equal to 251.5% and 96.2%, respectively. The sorption capacity follows the following order: pump oil < kerosene < naphtha. This can be attributed to the dynamic viscosity of the investigated oils [44]. The lower the dynamic viscosity, the higher the sorption capacity. Dynamic viscosity is a measure of a fluid’s resistance to deformation; thus, it is logical to surmise that pump oil with a higher dynamic viscosity (0.1231 Pa*s) has difficulty in entering the pores of the foam. On the other hand, oils with a lower resistance to deformation (dynamic viscosity of kerosene and naphtha, 0.0019 and 0.0012 Pa*s, respectively) can be easily sorbed by the composite foams. However, this behavior is also influenced by the oils’ high affinity with the foams.

**Figure 5:**
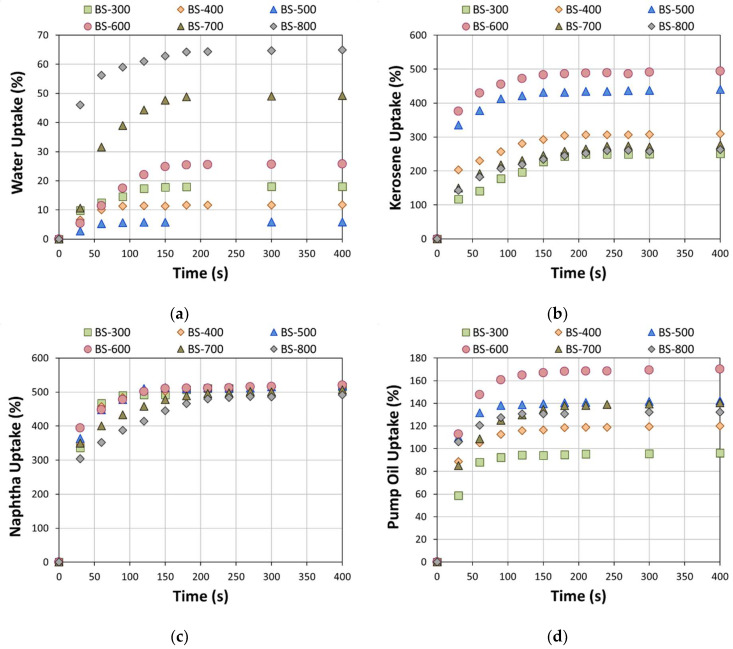
Sorption capacity (uptake) at increasing sorption time for all composite foams in (**a**) water, (**b**) kerosene, (**c**) naphtha, and (**d**) pump oil.

The sorption capacity (uptake) at increasing sorption time for the foams with varying bentonite filler in all liquids is shown in Figure 5. Some relevant information can be attained in order to assess the water uptake evolution during the sorption time of all of the foams (Figure 5a). All batches highlighted a progressive increase in uptake with increasing sorption time. A similar trend can be observed by evaluating the sorption capacity of the bentonite–PDMS foams in all other liquids (Figure 5b–d). Moreover, by increasing the treatment temperature, the bentonite releases structural water, which leads to a greater interaction between the sorbent surface and the oils. All kinds of bentonite treated at 400–600 °C can better improve the surface area. The structure of bentonite is not damaged, and has enough activity to facilitate the sorption of organic matter [45]. However, following an excessive heat treatment, the structure becomes compact [40] and no longer allows for an easy sorption process. In synergy with this, there is a progressive increase in foams’ pores as the treatment temperature of the bentonite increases (Table 2) due to a progressive reduction in the number of reactive sites in the sorbent, which enhances the foaming ratio in the silicone constituents. Due to these factors, an optimum sorbent performance has been found for the BS-500 and BS-600 formulations.

Bentonite clay is characterized by a surface negative charge, which attracts positively charged ions such as those found in oils and other impurities [46,47]. SEM images show that the bentonite is embedded in the silicone. However, it can be considered suitable to interact with oil, as the latter diffuses through the channels of the foam. At the same time, silicone foams contribute to the sorption capacity as a result of their intrinsic macro-porous structure that favor the oil diffusion in the channels, and repel water due to the high hydrophobic behavior of the PDMS matrix. This composite foam was tailored to trigger a synergistic interaction between the clay filler and oil, and the intrinsically water/oil selectivity of silicone-based macro-porous foams [35].

In order to have an adequate oil recovery capacity, as indicated above, the sorbent foams must be able to sorb a low water content and a high quantity of petroleum pollutants. This feature is an essential precondition to be able to offer an effective selective sorption of polluted oils dispersed in water. Regardless of the sorbed liquid, all foams have a rapid sorption kinetic. The curves show the stabilization of sorbate uptake at saturation after about 100–200 s. This allows us to hypothesize that it is possible to have an effective removal of the oils in relatively short immersion times.

**Figure 6:**
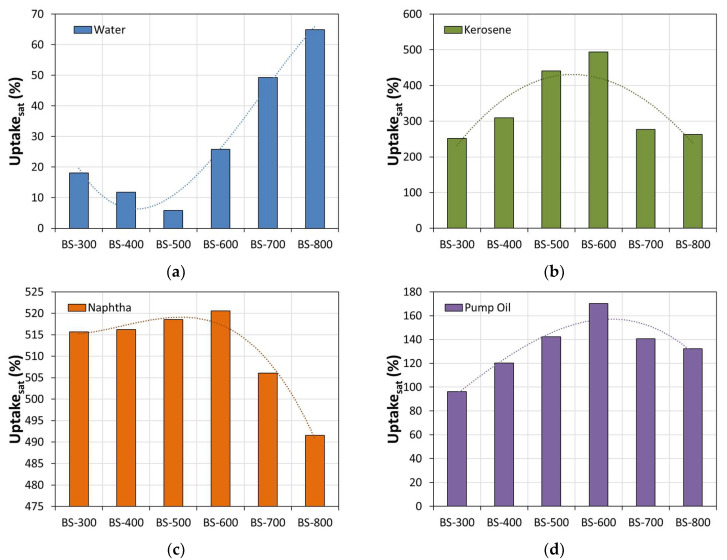
Uptake at saturation in (**a**) water, (**b**) kerosene, (**c**) naphtha and (**d**) pump oil for all bentonite–PDMS composite foams.

In order to better compare the sorption capacities of the foams in the different oils, Figure 6 shows the sorption values at saturation and for all of the sorbing foams in the different sorbed liquids investigated.

In this regard, a very high sorption capacity in water was observed for foams with bentonite treated at high temperature (BS-700 and BS-800). This value is at least 38% lower for other foams (BS-400). Evaluating the BS-300 foam, it is found that the composite material shows an acceptable water-repelling capacity, showing maximum sorption at a saturation of 18.1%. By increasing the temperature of the heat treatment on the bentonite (BS-400 and BS-500), an enhancement of the surface hydrophobic properties of the foam is detected. Indeed, the BS-500 composite foam exhibited a very low interaction with the water, providing a maximum sorption capacity at a saturation of about 5.9%. A further increase in temperature on the bentonite pretreatment leads to an increase in the hydrophilicity of the composite foam. This effect is increasingly noticeable the higher the temperature applied is.

This behavior can be attributed to two competing mechanisms that are able to enhance the hydrophilic or hydrophobic properties of the bentonite surface: the transformation of bentonite, which compacts at high treatment temperatures, thus losing its adsorbent character; and the larger size of the foam pores, caused by the favored foaming process. The larger pore size favors sorption towards a more viscous adsorbate (water and pump oil), which enters the pores and remains trapped, not being able to exit as easily as the less viscous oils [35]. Moreover, thermal treatment can affect the polarity of bentonite by altering its surface charge and functional groups. Furthermore, analyzing the uptake trend at saturation in oils, it is worth noting that the maximum oleophilic properties are found for composite foams made of bentonite thermally treated at intermediate temperatures (BS-500 and BS-600). In particular, the maximum sorption capacity is observed in kerosene and naphtha for the BS-600 batch with an uptake at saturation of 496.8% and 520.1%, respectively. The obtained results are comparable with those of other systems studied in the literature. Fe_2_O_3_@carbon core–shell nanoparticles prepared via the hydrothermal carbonization method developed by Kavil et al. [48] are capable of performing the instantaneous removal of 5 g g^−1^ of oil from the spillage with the assistance of an external magnet. Natural rubber studied by Songsaeng et al. [49] exhibits results comparable to those found in this study.

Thermally treated bentonite initially resulted in a better retention of oil spills. This behavior may be a result of the increased surface area and porous structure produced when the clay particles are heated, probably in conjunction with a change in the surface tension of the clay surface that stimulates its oleophilic nature. This allows a greater amount of oil to be sorbed as the pores act like miniscule traps for the oil particles. However, heat treatment at excessively high temperatures affects the performance of the materials and leads to a significant decrease in the sorption capacity. A range of 500–600 °C represents a transition temperature at which the maximum oil sorption capacity and minimum surface hydrophilicity are observed.

This confirms that the thermal treatment has been recognized as a cost-effective modification method for improving the clay minerals’ sorption capacity [50,51]. This behavior can be justified considering that when the temperature increases, water molecules located in the intracrystalline structure of bentonite can be selectively removed with different treatment temperatures [52]. Depending on the applied thermal treatment, the modification of the structure competes between dehydration and sintering conditions, which thus become critical factors affecting the sorption capacity [41,53]. The sorbed and interlayer water is completely removed at a temperature of about 400 °C [54].

At the same time, as previously mentioned, thermal treatment can affect the polarity of bentonite by altering its surface charge and functional groups. In a range between 400 and 800°, with the maximum rate being above 650 °C, the loss of dihydroxylation water takes place [55]. Therefore, thermal treatment can affect the functional groups of bentonite via the ihydroxylation and decomposition of some minerals. This can result in the loss of some functional groups, such as Si-OH, Al-OH, Mg-OH and CO_3_^2−^, and the formation of new ones, such as Si-O-Si, Al-O-Al and -OH [54].

However, at higher temperatures, the free volume in the clay structure is significantly reduced, thus limiting its sorption capacity. In fact, the surface area increases rapidly while the heating temperature rises to about 400–500 °C due to water dehydration, and then decreases progressively, caused by the dihydroxylation reaction, finally reaching zero at 900 °C due to decrystallization [54]. Therefore, at a molecular level, an intermediate thermal treatment could represent a crucial compromise to make the surface more oleophilic and hydrophobic.

Further information can be acquired by evaluating the ability of composite foams to adsorb hydrocarbons despite the presence of water. In this regard, an oil–water selectivity index has been defined as the ratio of the uptake at equilibrium in oil, Uptake_sat_(oil), (kerosene, naphtha and pump oil) versus the uptake at equilibrium in water, Uptake_sat_(water), according to the following formula:

(2) $$SI=\frac{{Uptake}_{sat}\left(oil\right)}{{Uptake}_{sat}\left(water\right)}$$

SI < 1 indicates that the sorption capacity of the foam is greater in water than in hydrocarbons. Conversely, SI > 1 indicates a greater sorption capacity in oils than in water. The higher the SI index, the more relevant the selective ability to adsorb oil is. Hence, e.g., during oil spill recovery in water, the composite foam, floating in the water, adsorbs oil preferentially over water, which has beneficial effects for the recovery procedures.

**Figure 7:**
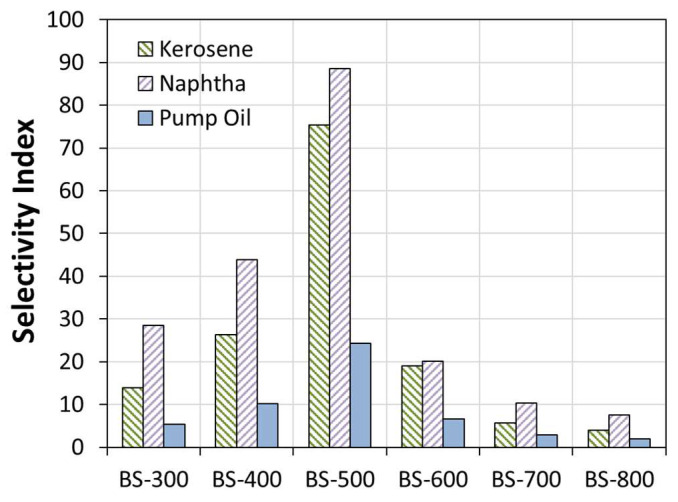
Oil–water selectivity index for all bentonite–PDMS composite foams.

Figure 7 compares the oil–water selectivity index for kerosene, naphtha and pump oil for all bentonite–PDMS composite foams. The results evidence that the thermally treated bentonite influences the interaction of the foam with water and oils. Evaluating the SI parameter experienced by the BS-300 sample, it is worth noting that, due to its acceptable hydrophobic behavior (water uptake at saturation for BS-300 is about 18.1%), the foam exhibits an SI index higher for kerosene and naphtha oils (13.9 and 28.6, respectively) than for pump oil (5.3).

Increasing the temperature of the thermal treatment, the bentonite acquires a marked hydrophobicity that leads to an evident oil–water selectivity. Indeed, the BS-500 foam experienced the highest SI values in all oils compared to all other batches. In particular, it reaches SI values of above 80 for light oils (kerosene and naphtha).

Bentonite foams have been identified as a highly efficient and effective solution for oil spill recovery, particularly for light oils. The surface tension, surface area and foaming properties of the foam enables it to float on top of the spilled oil; therefore, these materials could potentially act as a sorbent barrier that selectively traps the oil, thus eliminating its spread in the environment. Furthermore, the foam can also be easily recoverable due to its squeezing capacity [32].

SI in pump oil is about three times lower than that in other oils (SI 24.3 in pump oil for the BS-500 batch). Although this value is considerably higher than that found for the other batches, this index is not considered to be high enough for oil recovery applications.

Composite foams made of filler treated at higher temperatures (BS-600, BS-700 and BS-800) show a progressive decay in the oil–water selectivity index in all oils. In particular, evaluating the BS-800 batch, SI indices are always below 10, regardless of the analyzed oils. This behavior is due both to the increased surface hydrophilicity (water uptake approximately 65.0%) and to the ineffective sorption capacity found with hydrocarbons (see Figure 6).

The results show that the thermal treatment of bentonite influenced oil/water selectivity in oil spill recovery. Treatment at intermediate temperatures, such as 500 °C, results in the maximum SI index. At lower temperatures, the oil selectivity increases, while higher temperatures tend to significantly reduce the oil–water selectivity. The thermal treatment increases the interlayer spacing and cation exchange capacity, the latter of which is responsible for the solid’s selectivity.

The synergistic action of the foam and the functional filler helps to modify the sorption properties of the composite material. Thus, it is possible to assume that the bentonite modified according to the heat treatment has a key role in enhancing the oleophilicity. In synergy, the silicone foam has a dominant role on the hydrophobicity of the material itself, although this contribution is influenced by the foam morphology. An optimal value of the SI parameter was experimentally found in the BS-500 and BS-600 specimens.

Therefore, the heat treatment clearly affects the sorbent behavior of the clay, favoring the sorption of oils and highlighting a hydrophobicity at temperatures around 500 °C. A further increase does not benefit the material. The treatments at 500 °C, considered a medium temperature, improve the properties without damaging the structure and guarantee an economic advantage compared to more stringent treatments.

## 4. Conclusions

The present study focused on the synthesis and characterization of an efficient and cost-effective sorbent material for oil spill recovery. Bentonite clay filler, incorporated in a PDMS matrix, was the active sorbent material investigated. The clay microstructural characteristics and the macroscopic morphology of the sorbent material contributed to the development of an efficient sorbent system. The macro-porous composite was realized by means of a foaming process. The thermal treatment of bentonite was studied to increase the oleophilic properties and the hydrophobicity of the material. Temperatures from 300 °C to 800 °C were investigated. By analyzing XRD diffractograms and increasing the thermal treatment temperature, a dehydration of the hydrated bentonite was noted. In addition, the pore size of the realized foams increased with clay treatment temperature, decreasing the apparent density of the related composite sorbate. By investigating the sorption capacity of the realized foams in water and in pollutants oils (kerosene, naphtha and pump oil), a different behavior was evidenced. All batches highlighted a progressive increase in the sorption uptake with increasing sorption time. The samples treated at medium temperature (500–600 °C) exhibited the maximum sorption capacity at saturation in light oils, around 520%, in naphtha, for BS-500 and BS-600. At the same time, by increasing the treatment temperature to 800 °C, a higher hydrophilicity was found, decreasing the oil selectivity for the BS-800 sample (SI < 10). This behavior can be attributed to competing mechanisms responsible for the enhancement of the hydrophilic or hydrophobic properties to bentonite surface. The transformation of bentonite during thermal treatment at lower temperatures diminishes the intralayer space, eliminating the intralayer water molecules and increasing the surface area; at higher temperatures, in contrast, it compacts the clay, causing the loss of its sorbent character. At the same time, the larger dimensions of the foam pores, caused by the favored foaming process at a temperature higher than 600 °C, favor water sorption. Moreover, thermal treatment can affect the polarity of bentonite by altering its surface charge and functional groups. Due to these results, an optimum compromise has been reached for the BS-500 and BS-600 formulations. Further investigations are required to correlate the chemical characteristics of bentonite with oil–water affinity.

## Figures and Tables

**Table 1:** Bentonite–siloxane solution composition of composite foam (percent by weight).

Compound	Type	wt.%
PDMS	Siloxane	30.8%
PMHS	Siloxane	15.4%
Water	Solvent	6.2%
Ethanol	Solvent	9.2%
Sn(II)	Catalyst	7.6%
Bentonite	Filler	30.8%
Bentonite vs. foam [wt.%]		40.0%

**Table 2:** Average pore size (APS) and apparent density (ρ_a_) of the composite foam at varying filler typologies.

	APS [mm]	ρ_a_ [kg/m^3^]
BS-300	1.02	660.31
BS-400	1.36	539.52
BS-500	1.37	589.57
BS-600	1.40	565.35
BS-700	1.83	414.01
BS-800	2.18	361.81

## Data Availability

The data presented in this study are available upon request from the corresponding author.
